# A prospective study of symptoms, function, and medication use during acute illness in nursing home residents: design, rationale and cohort description

**DOI:** 10.1186/1471-2318-10-47

**Published:** 2010-07-14

**Authors:** William W Hung, Sophia Liu, Kenneth S Boockvar

**Affiliations:** 1 Center for the Study of Health Care Across Systems and Sites of Care, Department of Veteran Affairs HSR&D Service, James J Peters VA Medical Center, 130 West Kingsbridge Road, Bronx, NY 10468, USA; 2 Department of Geriatrics and Palliative Medicine, Mount Sinai School of Medicine, 1 Gustave L Levy Place, New York, NY 10029, USA; 3 Department of Anesthesiology, New York-Presbyterian/Columbia University Medical Center, 177 Fort Washington Avenue, New York, NY 10032, USA; 4 Research Institute on Aging, Jewish Home Lifecare, 120 W 106th Street, New York, NY 10025, USA

## Abstract

**Background:**

Nursing home residents are at high risk for developing acute illnesses. Compared with community dwelling adults, nursing home residents are often more frail, prone to multiple medical problems and symptoms, and are at higher risk for adverse outcomes from acute illnesses. In addition, because of polypharmacy and the high burden of chronic disease, nursing home residents are particularly vulnerable to disruptions in transitions of care such as medication interruptions in the setting of acute illness. In order to better estimate the effect of acute illness on nursing home residents, we have initiated a prospective cohort which will allow us to observe patterns of acute illnesses and the consequence of acute illnesses, including symptoms and function, among nursing home residents. We also aim to examine the patterns of medication interruption, and identify patient, provider and environmental factors that influence continuity of medication prescribing at different points of care transition.

**Methods:**

This is a prospective cohort of nursing home residents residing in two nursing homes in a metropolitan area. Baseline characteristics including age, gender, race, and comorbid conditions are recorded. Participants are followed longitudinally for a planned period of 3 years. We record acute illness incidence and characteristics, and measure symptoms including depression, pain, withdrawal symptoms, and function using standardized scales.

**Results:**

76 nursing home residents have been followed for a median of 666 days to date. At baseline, mean age of residents was 74.4 (± 11.9); 32% were female; 59% were white. The most common chronic conditions were dementia (41%), depression (38%), congestive heart failure (25%) and chronic obstructive lung disease (27%). Mean pain score was 4.7 (± 3.6) on a scale of 0 to 10; Geriatric Depression Scale (GDS-15) score was 5.2 (± 4.4). During follow up, 138 acute illness episodes were identified, for an incidence of 1.5 (SD 2.0) episodes per resident per year; 74% were managed in the nursing home and 26% managed in the acute care setting.

**Conclusion:**

In this report, we describe the conceptual model and methods of designing a longitudinal cohort to measure acute illness patterns and symptoms among nursing home residents, and describe the characteristics of our cohort at baseline. In our planned analysis, we will further estimate the effect of the use and interruption of medications on withdrawal and relapse symptoms and illness outcomes.

## Background

Nursing home residents are at high risk for developing acute illnesses [1,2]. Acute illnesses in older adults often are associated with complications, such as functional decline and death [3-5]. Although the consequences of acute illnesses and hospitalizations among community dwelling older adults are well described [3-5], less is known about the consequences of acute illnesses among nursing home residents. Compared with community dwelling adults, nursing home residents are often more frail, prone to multiple medical problems and symptoms, and are at higher risk for adverse outcomes from acute illnesses [1,2].

In the setting of acute illness, because of frailty and high burden of chronic disease, nursing home residents are particularly vulnerable to disruptions in transitions of care such as medication interruptions due to inadequate reconciliation [6-8]. In addition, interruption of medications which act on the central nervous system (CNS), including opioid analgesics, antidepressants and antipsychotic medications, confer a risk of adverse withdrawal events [9-13]. These medications are commonly used to manage pain, depression, psychosis and other symptoms among nursing home residents [9-13]. Interruptions of these medications, which act on the central nervous system, can lead to withdrawal syndromes, which often include distressing symptoms such as nausea, vomiting, anxiety, agitation, tremor and restlessness [14-16].

In order to better estimate the effect of acute illness on nursing home residents, we have initiated a study which will allow us to observe patterns of acute illnesses and the consequence of acute illnesses, including symptoms and function, among nursing home residents. We also aim to examine the patterns of CNS medication interruption, and identify patient, provider and environmental factors that influence continuity of medication prescribing at different points of care transition.

## Methods

### Study Model

**Figure 1:**
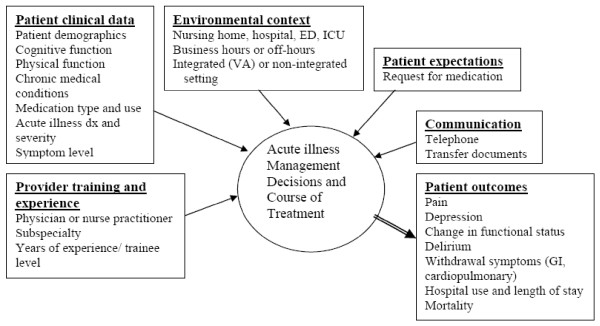
**Conceptual Framework: Factors influencing decision whether to continue CNS medication in acutely ill nursing home residents**.

We created a conceptual model adapted from the "Rapid Clinical Decision Making in Context" model [17], describing factors that may influence clinical decisions in nursing home residents in the setting of acute illness (AI). We included the following categories of factors as shown in Figure 1: provider knowledge and experience, patient clinical data, environmental context, patient expectations and communication. We hypothesized that influential factors vary during the episode with, for example, illness severity more influential at illness onset and hospital admission than at hospital discharge, and communication more influential at hospital admission and discharge than at illness onset. The main outcomes of acute illness in our model are those that are clinically important in nursing home patients such as function change, symptoms (pain, delirium, and medication withdrawal symptoms), and mortality.

### Study Design

The study is a prospective observational study of nursing home residents in 2 nursing homes in metropolitan New York--Jewish Home Lifecare (JHL), New York, NY and the James J. Peters VA (JJP VA) Community Living Center (CLC) in Bronx, NY. These nursing homes were chosen because during acute illness episodes, residents needing hospital care are referred predominantly to the Mount Sinai Hospital for JHL residents, and to the James J Peters VA Medical Center for JJP VA CLC residents; thus we are able to follow residents at these hospitals, if necessary, during their acute illnesses to monitor withdrawal of CNS medications and to measure outcomes.

### Eligibility Criteria

Residents are eligible to be enrolled in the study if they are receiving opioids, antidepressants, or antipsychotics on a routine basis for a duration of time considered to be a minimal therapeutic trial or in which tolerance develops. Because of the differences in pharmacokinetics and biologic effects among the 3 drug classes, a minimal therapeutic trial of 14, 30 and 7 days was selected for opioids, antidepressants, and antipsychotics respectively. We are including residents taking more than one medication in a class, or more than one class of medication if there are no known interactions between the medications. We are excluding residents receiving antidepressants who score fewer than 21 points on the Mini-Mental Status Examination [18] because the assessment of symptoms of depression, which is a main outcome, among those with more severe degrees of dementia, is less reliable. We are excluding residents who have an acute medical illness (defined below) at the time of screening and rescreening them for enrollment after it has resolved. Informed consent is obtained from residents or proxies. The study was approved by the Institutional Review Board at JHL and JJP VA Medical Center.

### Acute Illness Surveillance and Points of Care Transition

Acute illness surveillance is performed twice weekly at the nursing home through communication with nursing home nursing staff and medical providers using established clinical criteria [19] for incipient cases. The clinical criteria for acute illness are meant to be sensitive for all acute medical problems experienced by nursing home residents and include clinical symptoms such as chest pain, dyspnea, diarrhea, acute change in mental status; clinical signs such as persistent increase or decrease in blood pressure, fever or hypothermia; and abnormalities in laboratory values such as a drop in hematocrit > 5 points with signs of acute bleeding. We follow patients for 14 days after illness onset, and if applicable, an additional 14 days each after hospital admission and hospital discharge. During each follow-up interval we assess the following signs and symptoms by patient and staff interview in the nursing home or in the hospital: pain, delirium, and withdrawal symptoms including gastrointestinal and cardiopulmonary signs and symptoms three times weekly, and mood and behavior once weekly, using validated instruments adapted for this population described below.

### Measures

Baseline demographic information including age, gender, and race of each participant is collected. In addition, at baseline we collect information on chronic medical conditions and medication use through medical record abstraction, and on physical and cognitive function by interviews with patients, or proxies, and nursing home staff. Functional status is measured using items from the Minimum Data Set Activities of Daily Living Scale (MDS-ADL) adapted for interview with nursing home staff [20]. The MDS-ADL scale ranges from 0 to 6 with higher scores indicating poorer functional status. Cognitive function is measured using items from the Minimum Data Set Cognitive performance scale (MDS-CPS) [21] adapted for interview with nursing home staff and from the Mini Mental Status Examination (MMSE) [18]. Illness severity is measured using a physiologic measure, the Inpatient Physiologic Failure Score (IPFS) [22], which is adapted and validated for use in the elderly population. In the setting of acute illness, participants are assessed for delirium using the Confusion Assessment Method (CAM) [23].

Withdrawal symptoms from opiate withdrawal are measured using the Clinical Opiate Withdrawal Scale (COWS) [24], which contains measurements of several common signs and symptoms of withdrawal including tachycardia, sweating, restlessness, tremor, yawning, anxiety or irritability, gastrointestinal upset, pupil dilation, gooseflesh skin, running nose or tearing, and bone or joint aches. Scores on this scale vary from 0 to 48 with higher scores indicating more severe withdrawal. Withdrawal symptoms from antidepressants are measured using the Discontinuation Emergent Signs and Symptoms (DESS) scale [25]. We modified the scale to include only items within the proposed diagnostic criteria for Serotonin Reuptake Inhibitor syndrome [26], which includes the following symptoms--headache, insomnia, irritability, anxiety, fatigue, paresthesias, tremors, visual changes, dizziness, nausea, diarrhea, stomach cramps, chills, flushing and gait instability. We classify delirium as a separate complication from withdrawal, although we consider it consistent with withdrawal.

Relapse symptoms such as depression, psychosis and pain are measured using standardized instruments. Depression is measured using the Geriatric Depression Scale (GDS) [27], a 15-point scale for measuring depression in the elderly; a score of 5 or above indicates a positive screen for depression. Psychosis and disturbed behavior are measured using the Cohen Mansfield Agitation Inventory (CMAI) [28]. This scale contains 29 behaviors including verbal and physical behaviors, examples of which are repetitiveness, screaming, verbal aggression, and wandering. On the scale, these behaviors are measured on how frequently they are observed. Scores range from 29 to 103, with higher scores indicating more agitation. We used this scale as a measurement of the effect of antipsychotic cessation, because antipsychotic withdrawal has been associated with relapse symptoms [29], which can be reliably measured using this scale.

Pain is measured using a variety of measures based on whether the participant can reliably indicate their pain and severity. For participants who can indicate their pain, a modified Brief Pain Inventory (BPI) [30] is used, which measures the location and the severity of pain experienced currently and also in the past 24 hours. Participants are also asked to indicate how much their pain has interfered with their mood, sleep, walking ability and other activities. If the participant is unable to complete the BPI, a McGill Present Pain Intensity Scale [31] from 0 to 5 is attempted, and if unable to complete this, the participant is asked if they have pain currently (yes/no). If the participant is unable to provide answers regarding their pain, the checklist of nonverbal pain indicators (CNPI) [32] is used. Nursing staff are asked if they have observed any vocal complaints including verbal complaints and non-verbal sounds such as moans or groans, facial grimaces, restlessness, bracing, and rubbing during activity or rest.

**Figure 2:**
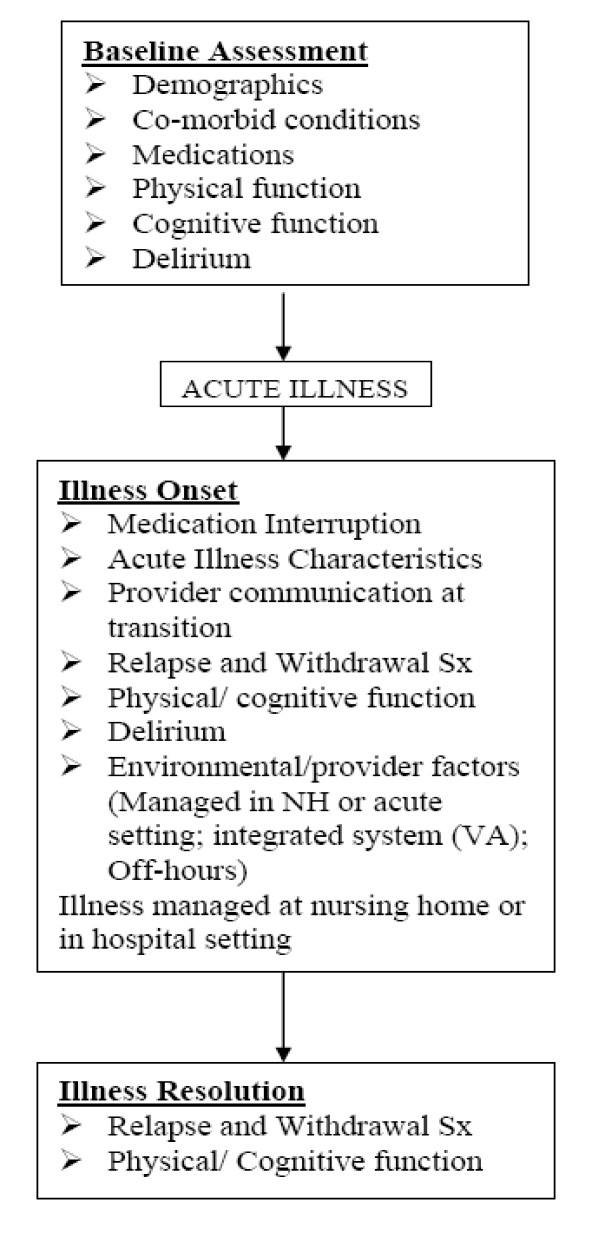
**Timeline of Assessments of Participants**.

Because symptoms of relapse and withdrawal can occur acutely and resolve in a short period of time, the pain, Clinical Opiate Withdrawal Scale (COWS), and Discontinuation Emergent Signs and Symptoms (DESS) scale are repeated three times a week during AI episodes to determine if withdrawal symptoms occurred. Functional status data are collected at the time of enrollment, subsequently at 3-month intervals and at the time of illness onset and 14 days after illness. Figure 2 outlines the timeline of our data collection during the study period.

### Statistical Analysis

Baseline demographic characteristics and symptoms measured on scales described above are summarized using descriptive statistics. Characteristics of acute illness including illness type and severity, are described. Planned analyses include estimation of the effect of acute illness on functional decline by comparing magnitude of functional change during periods with acute illness with periods without acute illness. Additional analyses include a description of the pattern of interruption of CNS medications during the acute illness and, to examine predictors of CNS medication interruption, estimation of a multivariable logistic regression model using occurrence of interruption in CNS medication as dependent variable, and patient, illness, and provider characteristics as independent variables. We will select independent variables to include in the model based on whether a factor is associated with the outcome in univariate analysis.

To examine the impact of CNS medication interruption on patient outcomes, we plan to estimate multivariable logistic regression models with pain, depression and disturbed behavior at the moderate or severe level as dependent variables and interruption in CNS medications as independent variable. Covariates will include patient and illness characteristics outlined in Figure 1. Furthermore, we plan to describe the effect of the interruption of CNS medications on hospital use, illness duration and functional outcomes, using similar multivariable regression models.

We also plan to compare proportions of patients who developed withdrawal symptoms who had medication interruption to those who did not have medication interruption. For the sample size of 200 AI episodes, we estimated that 20% of them would have an interruption of CNS medications. Assuming that the proportion who would have withdrawal symptoms without interruption of CNS medications (due to other causes such as the acute illness itself) to be 15%, this will provide 83% power (with an alpha of 0.05) to detect an absolute difference of 25% in the proportion with interruption of CNS medications who develops withdrawal symptoms (with a proportion of 40%). To estimate the effect of acute illness on function, we collected functional status data at the time of enrollment, subsequently at 3 month intervals, and at the time of illness onset and 14 days after illness. We plan to use a linear mixed model to account for such repeated measurements.

## Results

Baseline characteristics of study participants are summarized in Table 1. Overall, 76 residents have been recruited for the study, including 46 from JJP VA and 30 from JHL. Average age at enrollment was 69 years (SD 11) at VA and 80 (SD 9) at JHL. Overall 32% were female; however, this varied by site with 77% female at JHL and 2% female at JJP VA. Overall 25% were Black and 12% Hispanic. The most common chronic conditions were dementia (N = 31, 41%) and depression (N = 30, 38%). Other common conditions included congestive heart failure (25%), chronic obstructive lung disease (27%) and stroke (15%). Median duration of stay in the nursing home prior to enrollment was 153 days, with an interquartile range of 55 and 563 days. The mean number of hospitalization episodes in the year prior to enrollment was 1.2 episodes per resident (SD 1.1). Mean number of medications taken was 7.6 (SD 3.2). A quarter of study participants were taking antidepressants only, 17% were taking antipsychotic medications only, and 33% were taking opioid medications only. 25% of residents were taking more than one class of CNS medications; the most frequent combinations were an antidepressant and an opioid medication (14%), followed by a combination of an antidepressant and an antipsychotic medication (7%). Average function score on the MDS scale was 2.3 (SD 2.1), which corresponds to a functional status which requires limited assistance in ADL activities. Average GDS score was 5.2 (SD 4.4); 47% had a score of 5 or higher indicating a positive screen for depression. Average pain score was 4.7 (SD 3.6), which corresponds to moderate pain; and 62% of residents reported that they have moderate or severe pain. Average CMAI score was 35.8 (SD 8.1); 29% had a score of 39 or over, which indicates frequent and severe agitation.

**Table 1:** Baseline characteristics of nursing home residents in the cohort

	Total (n = 76)	JJP VA (n = 46)	JHL (N = 30)
Age (mean (SD))	74.4 (11.9)	69.4 (10.8)	82.0 (9.4)
			
Female	31.6	2.2	76.7
			
Race			
White	57.9	45.7	76.7
Black	29.0	43.5	6.7
Hispanic	11.8	10.9	13.3
			
Comorbid Conditions (%)			
CHF	25.0	17.4	36.7
COPD	27.6	32.6	20.0
Stroke	15.8	15.2	16.7
Dementia	40.8	21.7	70.0
Depression	38.2	37.0	26.7
Falls	52.6	41.3	70.0
			
Medications (total no. ± SD)	7.6 ± 3.2	8.0 ± 3.6	6.8 ± 2.6
			
CNS Medications (%)			
Antidepressants	25	22	30
Anti-psychotic medications	17	17	17
Opioids	33	30	37
Any combination of 2 or more	25	30	16
			
Duration of NH residence (Median, Inter Quartile Range)	153 (55,563)	74 (36, 233)	250 (107, 729)
			
Number of hospitalization in the year prior to enrollment ± SD	1.2 ± 1.1	1.5 ± 1.2	0.7 ± 0.5
			
Number of acute illness episodes (per resident per year)	1.5 ± 2.0	1.7 ± 2.2	1.0 ± 1.3
Function (MDS-ADL)	2.3 ± 2.1	2.2 ± 2.3	2.4 ± 2.0
Cognitive function (MDS-CPS)	1.1 ± 1.6	1.0 ± 1.8	1.3 ± 1.5
Depression (GDS-15)	5.2 ± 4.4	5.5 ± 4.4	4.8 ± 4.5
Pain (0-10 scale)	4.7 ± 3.6	5.5 ± 3.3	3.4 ± 3.7
Psychosis and disturbed behavior (CMAI)	35.8 ± 8.1	35.9 ± 7.9	35.7 ± 8.6

With a median follow up time of 666 days, to date we have observed a total of 138 acute illness episodes, for an incidence of 1.5 (SD 2.0) episodes per resident per year, 74% of which were managed in the nursing home setting. Characteristics of AI episodes are summarized in Table 2. Residents managed in the nursing home tended to be less ill, with a mean IPFS score of 1.5, compared with those managed in the hospital, with an IPFS of 5.3 (p < 0.001). The most common diagnoses of AI managed in the NH were urinary tract infection and cellulitis, whereas the most common diagnoses in the hospital setting were cardiac causes (such as congestive heart failure or myocardial infarction) and pneumonia.

**Table 2:** Characteristics of Acute Illness Episodes

	Managed at NH	Managed in Hospital
Number of Acute Illness Episodes	102 (74%)	36 (26%)
IPFS score (SD), Illness Severity	1.5 (2.5)	5.3 (4.2)
AI characteristics		
Urinary tract infection	32%	12%
Cellulitis	18%	4%
Pneumonia	6%	19%
Dehydration	5%	4%
Cardiac (MI/CHF/arrhythmia)	3%	27%
COPD	3%	8%
TIA/CVA	2%	4%
Sepsis	2%	12%
Other	37%	46%

## Discussion

In this paper we describe an ongoing longitudinal cohort study designed to measure symptoms and function change in nursing home patients with acute illness, to observe the continuity of CNS medication use for depression, pain and psychosis, and the consequence of CNS medication interruptions among nursing home residents in the setting of acute illness. Using our data thus far, we found that symptom burdens in our study cohort at baseline were high despite treatment. About two thirds of the study cohort had moderate or severe pain at baseline; close to half screened positive for depression; and 29% likely had clinically relevant agitation. Although our high rates may reflect our patient selection, it also likely reflects that symptoms persist despite treatment, and that nursing home residents with depression, pain and agitation may be under-treated. In addition, correlating with prior research [1], we confirmed that the majority of acute illnesses (74%) among nursing home residents were treated in the nursing home rather than in the acute care setting. Not surprisingly, acute illnesses of higher severity and spectrum were treated in the hospital compared with those treated in the nursing home setting. We have planned further analysis to address several other research questions pertaining to clinical decision making, health services and outcomes. Table 3 highlights the research questions we plan to address using our cohort design.

**Table 3:** List of research questions potentially addressed in this cohort study

A. Health services and quality of care
1. Among nursing home residents, what is the impact of acute illness, managed at the nursing home or in the hospital, on patient outcomes including function and mortality?
2. What is the impact of acute illness on symptoms such as pain, depression, agitation?
3. What is the effect of acute illness on functional status, which is a publicly-reported measure of nursing home quality?
4. How often are CNS medications interrupted during care transition periods and what are the factors affecting the pattern of medication interruption?
**B. Clinical decision**
1. What is the balance of benefit and risk of holding opiates and other CNS medications during acute illness episodes?

**C. Epidemiology**
1. What is the longitudinal symptom burden, including pain, depression, agitation and others, among nursing home residents?

Our study also highlights some methodological features which may be utilized by other investigators designing similar studies in this population. In order to determine the impact of acute illness, the research team actively surveys for the occurrence of acute illness. Previous studies relying on medical records or referrals from providers are likely to miss acute illnesses which are less severe or are treated in the nursing home setting. Because reliable reports of symptoms such as pain, delirium, and withdrawal symptoms require real-time ascertainment, the active surveillance by the research team is essential to capture acute illnesses and symptoms prospectively. In addition, because transitions of care across multiple settings occur in nursing home residents experiencing acute illnesses, it is essential for the research team to conduct symptom monitoring in these different settings. Symptoms are assessed frequently in this study because symptoms of withdrawal can occur acutely and resolve in a short period of time.

Furthermore, the choice of the two nursing homes in our study also allows us to examine the effect of an integrated medical system with electronic medical record sharing on medication interruption and continuity of care. Medication and treatment information is readily available across nursing home and hospital settings through an electronic medical record system in the VA-based nursing home. On the other hand, JHL does not have an integrated electronic system. Therefore, providers rely on traditional methods of communication using transition documents or telephone communication.

A limitation of our study is that our examination of different levels of factors affecting medication interruption is limited by the proportion of AI with medication interruption. The analysis of the effects of multiple factors may require a larger sample size. However, we will likely be able to observe clinically important effects that are of larger magnitude. In addition, considering the two cohorts included in the sample, there were significant differences in the characteristics of the VA cohort compared with the JHL cohort. The VA cohort was younger, more likely to be male, black and less likely to have dementia. The different characteristics of the VA cohort may limit generalizability to community nursing homes, but the inclusion of the VA cohort also help complement the gender and racial makeup of the study sample.

## Conclusions

In this paper, we described the methods of our study in detail and our cohort characteristics. The aim of our study is to inform nursing home physicians the effect of acute illness on symptoms, function change and medication use in nursing home residents. Considering that nursing home residents are likely to have high symptom burden, and to be vulnerable to acute illness and to disruptions in care across care settings, the study has the potential to improve the care of nursing home residents across care settings.

## Competing interests

The authors declare that they have no competing interests.

## Authors' contributions

WWH contributed to the conception and design, collection of data, analysis and interpretation, and preparation of the manuscript. SL contributed to the conception and design, collection of data, and preparation of the manuscript. KSB contributed to the conception and design, data collection, analysis and interpretation and preparation of the manuscript. All authors read and approved the final manuscript.

## Pre-publication history

The pre-publication history for this paper can be accessed here:

http://www.biomedcentral.com/1471-2318/10/47/prepub
